# Regulation of Gene Expression in *Neurospora crassa* with a Copper Responsive Promoter

**DOI:** 10.1534/g3.113.008821

**Published:** 2013-10-18

**Authors:** Teresa M. Lamb, Justin Vickery, Deborah Bell-Pedersen

**Affiliations:** Department of Biology, Texas A&M University, College Station, Texas 77843

**Keywords:** gene function, protein overexpression, regulation of essential genes, bathocuproinedisulfonic acid, copper sulfate (CuSO_4_)

## Abstract

Precise control of gene expression is a powerful method to elucidate biological function, and protein overexpression is an important tool for industry and biochemistry. Expression of the *Neurospora crassa tcu-1* gene (NCU00830), encoding a high-affinity copper transporter, is tightly controlled by copper availability. Excess copper represses, and copper depletion, via the use of a copper chelator, activates expression. The kinetics of induction and repression of *tcu-1* are rapid, and the effects are long lived. We constructed a plasmid carrying the *bar* gene (for glufosinate selection) fused to the *tcu-1* promoter. This plasmid permits the generation of DNA fragments that can direct integration of P*_tcu-1_* into any desired locus. We use this strategy to integrate P*_tcu-1_* in front of *wc-1*, a circadian oscillator and photoreceptor gene. The addition of excess copper to the P*_tcu-1_*::*wc-1* strain phenocopies a *Δwc-1* strain, and the addition of the copper chelator, bathocuproinedisulfonic acid, phenocopies a *wc-1* overexpression strain. To test whether copper repression can recapitulate the loss of viability that an essential gene knockout causes, we placed P*_tcu-1_* upstream of the essential gene, *hpt-1*. The addition of excess copper drastically reduced the growth rate as expected. Thus, this strategy will be useful to probe the biological function of any *N. crassa* gene through controlled expression.

Genome sequencing of many organisms has greatly advanced our understanding of biology, but there is still much to learn about gene function. Genes without a known function comprise ~40% of both the human and the *Neurospora crassa* genomes (Galagan *et al.* 2003; Venter *et al.* 2001). Even *Saccharomyces cerevisiae*, the first eukaryote with a completely sequenced genome, still has roughly 1000 genes that are uncharacterized (Pena-Castillo and Hughes 2007). Discovering the function of these genes is an important goal for future advances in biological understanding.

The ability to control gene expression by both activation and repression is a useful method to discover biological function. Furthermore, biochemical studies and industrial production of proteins can be accelerated by the ability to generate large amounts of protein. Fungal model systems have been incredibly useful in large-scale protein production, as well as in determining biological functions of genes (Borkovich *et al.* 2004; Nevalainen *et al.* 2005; Winzeler *et al.* 1999). In *N. crassa*, the *qa-2* promoter provides a control system that activates gene expression in the presence of quinic acid and low sugar concentrations and turns down gene expression at low quinic acid or high sugar concentrations (Campbell *et al.* 1994; Giles *et al.* 1985). The *qa-2* promoter is useful, but not ideal, for gene expression manipulation because it is highly influenced by the nutritional state of the culture, and expression levels are generally not greatly enhanced. The copper metallothionein promoter (*cmt*) has been used as a copper-inducible promoter (Kupper *et al.* 1990; Schilling *et al.* 1992), but this promoter has not been studied for repression. The *vvd* promoter has recently been used to drive light-regulated induction (Hurley *et al.* 2012), but problems arise with this method when light-regulated processes are studied. Furthermore, overexpression of proteins in this system is transient unless generated in a *Δvvd* background. Other promoters also have been used to drive exogenous expression in *N. crassa*, but they are either constitutive or controlled by nutritional and/or developmental state (*tub-1*, *ccg-1/grg-1*; Honda and Selker 2009; Nakano *et al.* 1993).

Copper is an essential cofactor for many enzymes in the cell, but excess copper can also be toxic (De Freitas *et al.* 2003; Thiele 2003). Organisms have developed regulatory mechanisms that are sensitive to environmental copper levels to control the uptake of copper (De Freitas *et al.* 2003). Three Neurospora copper transporter genes have been shown to be responsive to copper availability at multiple developmental stages (Korripally *et al.* 2010). Studies in the yeast *Saccharomyces cerevisiae* have shown that only a small portion of the genome is regulated by copper availability, which makes copper a good candidate molecule for exogenous control of gene expression (Gross *et al.* 2000; Rustici *et al.* 2007; van Bakel *et al.* 2005). Given the success of using the high-affinity copper transporter promoter in *Schizosaccharomyces pombe* and *Cryptococcus neoformans* to drive heterologous gene expression (Bellemare *et al.* 2001; Ory *et al.* 2004), we examined the use of this promoter in *N. crassa*.

First, we examined the expression of *tcu-1* (NCU00830), because this gene is most similar to the *S. pombe* and *C. neoformans* high-affinity copper genes. As expected, *tcu-1* responded to changes in external copper levels (Korripally *et al.* 2010), and we defined the kinetics of that response. Next, we devised a customizable strategy that replaces, or inserts, the copper-responsive *tcu-1* promoter (*P_tcu-1_*) into the 5′ region of a target gene with the following advantages: (1) tunable activation and repression using the same strain; (2) small (500-bp) regions of the gene are used for integration at genomic locus, bypassing the need to clone large genes; (3) expression is regulated by simply adding CuSO_4_ or BCS; (4) there is no need to alter the sugar content; and (5) one can simultaneously integrate *P_tcu-1_* while deleting target gene control regions. Finally, as proof of concept, we used this strategy to control expression of two genes.

## Materials and Methods

### Culture conditions

*N. crassa* (FGSC #2489, 74-OR23-IV, *mat a*, called “WT”) was grown in 75 mL 1× Vogels salts, 2% glucose, 0.5% arginine, pH 6.0 (unless otherwise noted) shaking cultures inoculated with mycelial discs cut from mats grown in the same media (McCluskey *et al.* 2010; Vogel 1956). This standard media contains 50 µM CuSO_4_. After 24 hr of growth, cultures were treated with CuSO_4_ (C7631; Sigma-Aldrich) and/or bathocuproinedisulfonic acid (BCS, B1125; Sigma-Aldrich) as described. The concentration of CuSO_4_ indicated in each experiment is the final total concentration. For the specificity experiment, cultures were treated with 10 mM quinic acid (138622; Sigma-Aldrich), 1 µg/mL fludioxonil (46102; Fluka), 0.4 M NaCl, 10 mM hydrogen peroxide (216763; Sigma-Aldrich), 0.05% sodium dodecyl sulfate (SDS, L4390; Sigma-Aldrich), 1× Westergaard’s medium (Westergaard and Mitchell 1947), or placed on a moist paper and exposed to the air.

To examine the effect of P*_tcu-1_*-driven WC-1 on circadian rhythms, race tubes containing 1× Vogels salt, 0.1% glucose, 0.17% arginine, 50 μg/mL biotin, and 1.5% agar with CuSO_4_ or BCS added before autoclaving were inoculated with fresh conidia, grown for 1 d at 30° in constant light (LL), and then shifted to constant darkness (DD) at 25°. The growth front was marked under a red safelight every 24 hr subsequently. The period of the developmental rhythm was determined as previously described (Dunlap and Loros 2005). We also determined the percent of linear hyphal growth (as opposed to aerial hyphae with conidial development) during the course of the day by measuring the average distance of “clear” hyphal growth in a day and dividing by the average total linear growth in that day.

To examine the effect of P*_tcu-1_*-driven histidine phosphotransferase (HPT-1) on growth rate, race tubes containing 1× Vogels salts, 2% glucose, 0.5% arginine, 50 μg/mL biotin, and 1.5% agar with CuSO_4_ or BCS added before autoclaving were inoculated with ~7-d-old conidia and grown for 3 d at 30° in LL.

### Construction of the pCR blunt *bar*::P*_tcu-1_* plasmid

The *Streptomyces hygroscopicus bar* gene conferring resistance to glufosinate (Avalos *et al.* 1989) was amplified by polymerase chain reaction (PCR) from pBAR-GEM 7.2 (Pall and Brunelli 1993) using primers Bar-BstB1-F (5′ TTCGAAGTCGACAGAAGATGATATTG 3′) and Bar-BstB1-R (5′ TTCGAAGAACCGGCAGGCTGAAGTCC 3′). The resulting 912-bp DNA fragment was ligated into pCR blunt (Invitrogen, Carlsbad, CA) generating plasmid pJV1. The 1690-bp DNA fragment containing the *tcu-1* promoter was generated by PCR on wild-type (WT) genomic DNA using primers P_tcu-1_ F-*Not*I (5′ TTTGCGGCCGCGATGGGATAGAGAGAATGGC 3′) and P_tcu-1_ R *Apa*I (5′ TTTGGGCCCGGTTGGGGATGTGTGTGC 3′). The PCR product was cut with *Not*I and *Apa*I, and ligated into pJV1 digested with the same enzymes, creating pCR blunt *bar*::P*_tcu-1_* (plasmid and sequence deposited at the Fungal Genetic Stock Center).

### Strains

To generate P*_tcu-1_*-driven WC-1 and HPT-1 cell lines, 5′ and 3′ integrating fragments of DNA were constructed as described in the *Results*, and ~50 ng of each were transformed by electroporation into FGSC 1858 (*ras-1^bd^*, *mat A*, called P*_wc-1_wc-1*), or DBP 1202 (*ras-1^bd^*, *mat a*, *hpt-1*::*FLAG*::*hph*, *rrg-1*::*HA*::*hph*, called P*_hpt-1_hpt-1*) strains, respectively (McCluskey *et al.* 2010). For *hpt-1*, the dual-tagged strain was generated for future studies to investigate interactions between HPT-1 and response regulator-1 (RRG-1). Colonies were selected on 250 µg/mL glufosinate ammonium (G596950; Toronto Research Chemicals)-containing transformation plates, and proper integration was confirmed as discussed in the *Results*. Homokaryons were obtained by crossing to FGSC 1859 (*ras-1^bd^*, *mat a*), creating DBP 1573 (*bar*::P*_tcu-1_*::*wc-1*, *ras-1^bd^*, *mat a*), subsequently called the P*_tcu-1_wc-1* strain, or by filtration (Ebbole and Sachs 1990), creating DBP 1660 (*bar*::P*_tcu-1_*::*hpt-1*::*FLAG*::*hph*, *ras-1^bd^*, *mat a*, *rrg-1*::*HA*::*hph*), subsequently called the P*_tcu-1_hpt-1* strain.

### Protein extraction and Western blots

*N. crassa* tissue was blotted dry, frozen, and subsequently ground under liquid nitrogen. Proteins were extracted from ground tissue (Garceau *et al.* 1997), and 100 µg of total protein was run on standard SDS polyacrylamide electrophoresis gels, and electro-transferred to polyvinylidene difluoride membranes (IPVH00010; Millipore). WC-1 protein was bound with primary mouse monoclonal anti-WC-1 antibody (diluted 1:200, a gift from M. Brunner; Gorl *et al.* 2001), recognized with a goat antimouse horseradish peroxide−conjugated secondary antibody (diluted 1:10,000, 170-6516; BioRad), and visualized with Super Signal Femto Maximum Sensitivity Substrate (34095; Thermo Scientific, Waltham, MA). HPT-1-FLAG was bound with primary DYKDDDDK Tag Antibody (diluted 1:1000, 2368; Cell Signaling Technology), recognized with goat antirabbit horseradish peroxide−conjugated antibody (diluted 1:20,000, 170-6515, Bio Rad), and visualized with a chemiluminescence solution (1.25 mM Luminol [A8511; Sigma-Aldrich], 0.2 mM p-coumaric acid [C9008; Sigma-Aldrich], 100 mM Tris-Cl, pH 8.5, and freshly added hydrogen peroxide [diluted 1:3333, 216763; Sigma-Aldrich]). After detecting via Western blotting, membranes were stained with an amido black solution (0.1% amido black [N3393; Sigma-Aldrich], 10% acetic acid, 25% isopropanol) to reveal all proteins as an indication of protein transfer.

### RNA extraction and Northern blots

RNA was extracted and purified from ground tissue, and 10 µg were run on denaturing formaldehyde gels (Bell-Pedersen *et al.* 1996). Gels were blotted to Nitro Pure membranes (#WP4HY00010; GE), and hybridized with gene specific RNA-probes labeled with [α-^32^P]-UTP (BLU507H250UC; PerkinElmer).

## Results

### *N. crassa tcu-1* gene expression is controlled by copper availability

We examined whether the high-affinity copper transporter (*tcu-1*, NCU00830) is regulated by copper availability. WT *N. crassa* was grown in liquid media with copper sulfate (50−250 µM), or a copper chelator (BCS, 50−250 µM) and harvested after 8 hr. The levels of *tcu-1* and control *actin* mRNAs were examined. Supporting Information, Figure S1 shows that although *actin* expression was unaffected by the abundance of copper, the endogenous *tcu-1* message was highly responsive to copper availability, with a 40-fold expression range under these conditions. Excess copper turned down *tcu-1* expression whereas increasing concentrations of the copper chelator, BCS, induced *tcu-1* expression, as predicted (Korripally *et al.* 2010).

### The kinetics of *tcu-1* activation depend on the copper concentration, and the kinetics of turn off are very rapid

**Figure 1:**
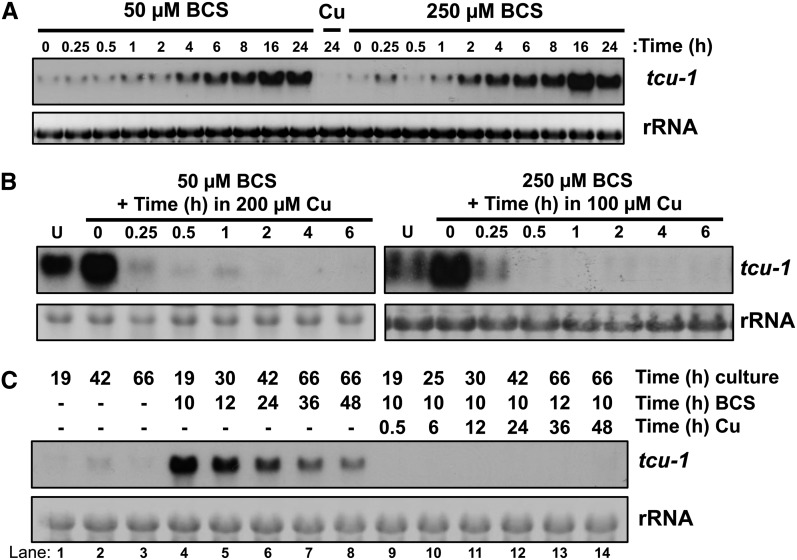
The kinetics of *tcu-1* gene expression responses to Cu/BCS in a WT strain. (A) Levels of endogenous *tcu-1* mRNA at the indicated times after the addition of 50 or 250 µM BCS or 250 µM CuSO_4_ (Cu) were determined by Northern analysis. Ethidium bromide staining of the rRNA shows even loading in all panels. Quantitation of two independent experiments is shown in Figure S2. (B) Levels of *tcu-1* mRNA were examined in untreated (U) or BCS treated (either 50 or 250 µM) cultures that were subsequently treated with 200 or 100 µM CuSO_4,_ respectively, for the indicated times by Northern analysis. (C) Induction and repression of *tcu-1* are long-lasting. Cultures grown for the indicated times were either untreated (lanes 1−3), treated with 200 µM BCS for the indicated times (lanes 4−14), or treated with 200 µM BCS and then subsequently treated with 250 µM CuSO_4_ for the indicated times (lanes 9−14). Levels of *tcu-1* mRNA were determined by Northern analysis. Two independent experiments were analyzed with similar results.

We investigated *tcu-1* expression at several time points after the addition of 50 µM and 250 µM BCS in a WT strain. Figure 1A shows that *tcu-1* was induced 4 hr after the addition of 50 µM BCS and 2 hr after the addition of 250 µM BCS. Induction of *tcu-1* was quantitated in two independent experiments, and the average expression level was plotted in Figure S2. These data shows that the concentration of copper chelator affected the kinetics of induction. Maximal levels of induction occurred between 8 and 24 hr, and expression remained high at 24 hr after addition of either concentration of BCS.

To study the effect of gene turn down/off, it is useful to have rapid and concentration insensitive kinetics. To test whether the *tcu-1* promoter fulfills this criterion, we activated *tcu-1* expression with either 50 µM BCS or 250 µM BCS for 4 hr, added CuSO_4_ at 200 µM or 100 µM respectively, and then analyzed the effect on *tcu-1* message over time in a WT strain. Figure 1B shows that turn off of *tcu-1* was robust in both experiments, with nearly all expression extinguished by 0.25 hr, and expression remained reliably low for at least 6 hr.

To examine how long *tcu-1* expression is either activated by BCS or repressed by Cu, we treated cultures with 200 µM BCS for various times with or without the subsequent addition of 250 µM CuSO_4_ (Figure 1C). *tcu-1* expression is quite low in the untreated samples independent of the culture time. Treatment with BCS for 10 hr maximally induced *tcu-1*, with longer treatments yielding lower induction. However, even after 48 hr in BCS, *tcu-1* is still greatly induced over the untreated samples. All samples treated with BCS first, and then treated with Cu for different amounts of time, maintained repression of *tcu-1*. Thus, induction of *tcu-1* by BCS or repression of *tcu-1* by CuSO_4_ can last for at least 2 days.

### Copper control of *tcu-1* gene expression is highly specific

**Figure 2:**
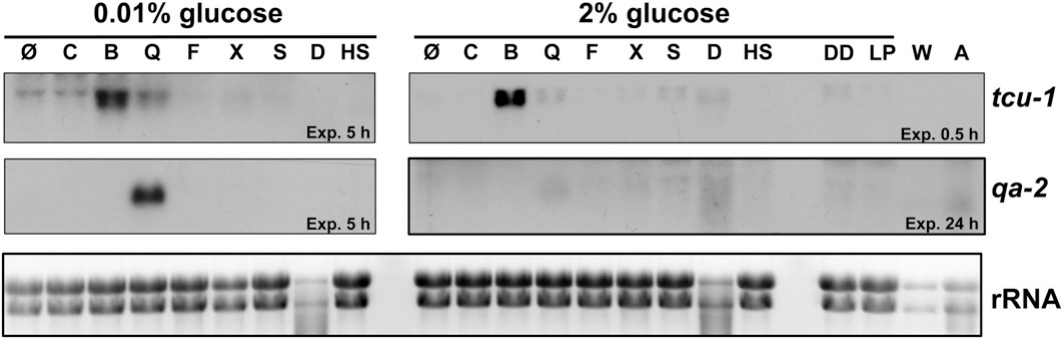
Effects of stress and nutrition on copper and quinic acid−controlled gene expression in a WT strain. The levels of *tcu-1* and *qa-2* mRNA were examined by Northern analysis in control untreated (Ø), 250 µM CuSO_4_ (C), 250 µM BCS (B), 10 mM quinic acid (Q), 1 µg/mL fludioxonil (F), 10 mM H_2_O_2_ (oxidative stress, X), 0.4M NaCl (salt, S), 0.05% SDS (detergent, D), and 30 min 42°C heat shock (HS) in 0.01% and 2% glucose conditions, as well as in 24-hr DD, a 30 min-light pulse (LP), Westergaard’s medium (W), and air exposure (A). The exposure times of the membranes with film are indicated for each blot. Ethidium bromide staining of the rRNA is shown in the bottom panel.

Ideally, a promoter used to drive controlled gene expression would respond primarily to the specific signal (in this case copper availability) and not to other external factors. To determine whether the *tcu-1* promoter fulfills this criterion, we tested the effects of media composition and various stresses on the native expression of *tcu-1* in a WT strain. We compared the expression of both *tcu-1* and the quinic acid responsive gene, *qa-2*, under these conditions. Figure 2 shows that in 2% glucose growth conditions, BCS strongly induced *tcu-1*. Osmotic, oxidative, heat, and cell wall stresses as well as growth in Westergaard’s medium, which stimulates sexual development, and air exposure, which stimulates conidiation, only weakly affected the expression of *tcu-1*. As predicted, *qa-2* was not induced in 2% glucose growth conditions, even when the inducer quinic acid was provided (Giles *et al.* 1985). In 0.01% glucose conditions *tcu-1* was still induced by BCS. There was weak induction of *tcu-1* by quinic acid, most likely resulting from the acidic environment inhibiting copper import, and thus depleting copper (Blackwell *et al.* 1995). As expected, *qa-2* was strongly induced by quinic acid in 0.01% glucose conditions (Giles *et al.* 1985). Loading of the RNA samples was uniform as shown in the bottom panel, with the exceptions that the detergent treated samples were somewhat degraded, and the Westergaards and air treated samples were low. These data indicated that *tcu-1* was specifically controlled by copper availability, and was generally insensitive to other tested perturbations. In addition previous studies have shown that *tcu-1* gene expression is independent of glucose, light, or circadian clock control (Lewis *et al.* 2002; Smith *et al.* 2010; Xie *et al.* 2004). Thus, the *tcu-1* promoter can be used for copper-controlled exogenous gene expression in both low- and high-glucose conditions and under many environmental stress conditions.

### A PCR-based strategy to integrate *P_tcu-1_* in front of any target gene

**Figure 3:**
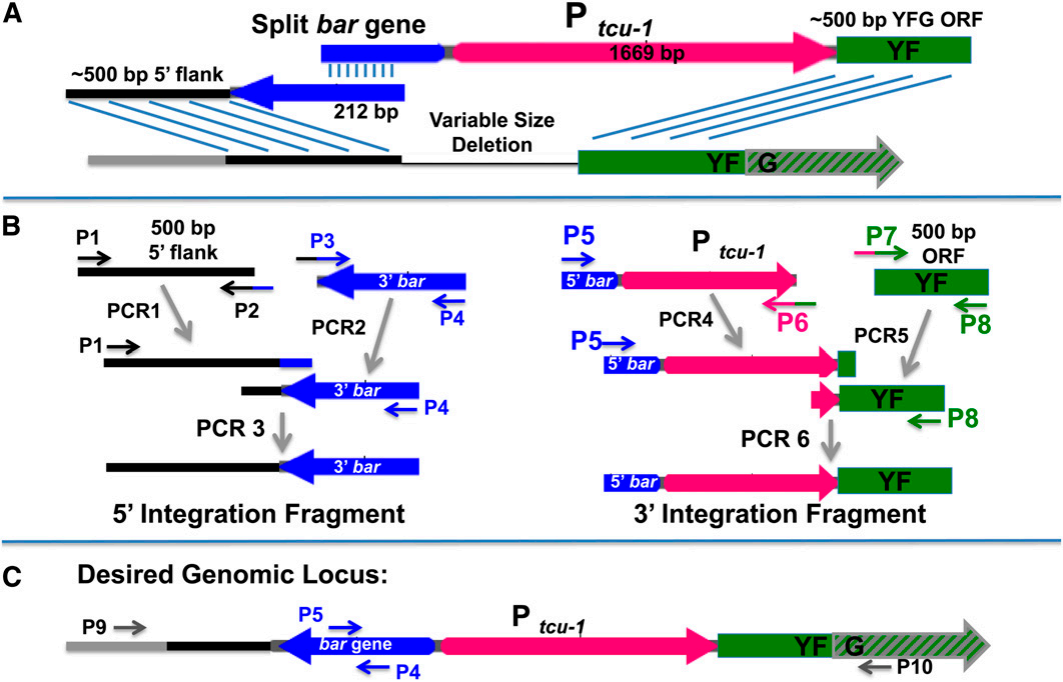
Scheme for integrating P*_tcu-1_* to a target genomic locus. (A) An overview of the scheme shows integration of two DNA fragments containing homology to the 5′ UTR of the target gene, the bar gene, the copper transporter promoter, and finally homology to the ORF of the target gene. Three recombination events (as denoted by parallel lines) are required to properly integrate the two fragments. (B) Production of the two integration fragments is described in the results. (C) The final desired genomic locus can be confirmed by PCR using primers P9 and P4 to validate correct 5′ integration, and P5 and P10 to validate correct 3′ integration. YFG, your favorite gene.

Figure 3 diagrams the strategy for integrating *P_tcu-1_* in front of any gene. This strategy is similar to the split marker integration scheme introduced (Catlett *et al.* 2003) and further explored by the *N. crassa* gene knockout project (Colot *et al.* 2006). The design of the required primers is shown in Table 1. Two PCR-generated fragments are cotransformed into *N. crassa* that can integrate the copper promoter into the desired genomic locus (Figure 3A). Transformants are selected by virtue of their resistance to the drug glufosinate upon recombination of the *bar* gene (Avalos *et al.* 1989).

**Table 1:** Primer design

Primer Name	5′-End Tail*^a^*	3′-End Homology
ORF-specific P1 F		F-strand promoter (~ −1022 to −1000)*^b^*
ORF-specific P2 R	*bar*: ttaggtcgac	R-strand promoter (~ −500 to −522)*^c^*
ORF-specific P3 F	F-strand promoter (~ −510 to −500)	*bar:* gtcgacctaaatctcggtgac
Universal P4 R		*bar:* atcgtcaaccactacatcgaga
Universal P5 F		*bar:* ggagacgtacacggtcgact
ORF-specific P6 R	R-strand YFG ORF (+10 to +1)	R Promoter *tcu-1:* GGTTGGGGATGTGTGTGCGA
ORF-specific P7 F	F promoter *tcu-1:* ATCCCCAACC	F strand YFG ORF (+1 to +20)
ORF-specific P8 R		R-strand YFG ORF (~ −522 to +500)
ORF-specific P9 F		F-strand promoter (~ −1050 to −1030)
ORF-specific P10 R		R-strand YFG ORF (∼ +550 to +530)

ORF, open reading frame; YFG = “your favorite gene.”

a Each primer is composed of a 5′ end tail (if needed) fused to the 3′ end homology sequence and is listed in the 5′ to 3′ direction. The gene region that the sequence corresponds to is indicated, followed by the sequence. For gene specific sequences the recommended region to obtain the sequence is listed.

b The numbers denote the recommended distance in bp from the start codon, with negative numbers upstream and positive numbers downstream. If known control sequences in your target must be deleted, then these numbers will need to be altered accordingly.

c Use of this position in the 5′ UTR will delete 500 bp upstream of the ATG.

Construction of each PCR fragment for integration depends on a two-step process, as shown in Figure 3B. PCR1 with primers P1 and P2 amplifies 500 bp of the 5′ flank of the target gene from genomic DNA. PCR2 with primers P3 and P4 amplifies the 3′ end of the *bar* gene from the plasmid pCR blunt *bar* P*_tcu-1_*. PCR3 with primers P1 and P4 on PCR1 and PCR2 templates generates the 5′ integration fragment. PCR4 with primers P5 and P6 amplifies the 5′ end of the *bar* gene fused to the *tcu-1* promoter from the plasmid pCRblunt *bar* P*_tcu-1_*. PCR5 with primers P7 and P8 on genomic DNA amplifies 500 bp of the target gene ORF. PCR 6 with primers P5 and P8 on PCR4 and PCR5 templates generates the 3′ integration fragment. Proper integration at the 5′ and 3′ ends of the genomic locus can be verified by PCR with use of the primer sets P9 and P4 and P5 and P10, respectively (Figure 3C).

### Copper control of a P*_tcu-1_wc-1* strain recapitulates deletion and overexpression phenotypes

As proof of principle, we chose to integrate P*_tcu-1_* into the *wc-1* genomic locus for two reasons. First, WC-1 protein levels can be easily monitored by Western blot (Gorl *et al.* 2001). Second, the phenotypes of WC-1 knockout *(Δwc-1*) and overexpression strains (P*_qa-2_*::*wc-1*) have been previously published (Cheng *et al.* 2001; Lee *et al.* 2003), thus providing predictable outcomes for P*_tcu-1_* control of WC-1.

The primers used to create *bar*::P*_tcu-1_*::*wc-1* integrating fragments are listed (Table S1). A defined 1.5-kb region of the *wc-1* promoter drives clock-controlled expression (Froehlich *et al.* 2002, 2003); therefore primers (WC-1 P1 and WC-1 P2) were designed to delete the clock control region. In this way, the desired genomic locus should only be under P*_tcu-1_* control. PCR products 1 through 6 were generated and the expected fragment sizes were confirmed (Figure S3A). PCR products 3 and 6 were cotransformed into *N. crassa*, and glufosinate-resistant colonies were examined for proper integration. Of nine glufosinate-resistant heterokaryotic clones, three (1, 7, and 9) displayed the proper 5′ genomic structure as indicated by the production of the expected 1.5 kb-DNA fragment using WC-1 P9F and P4R primers in a PCR on genomic DNA (Figure S3B). Clones 1 and 7 also displayed the proper 3′ genomic structure as indicated by the production of the expected 3.1-kb DNA fragment using P5F and WC-1 P10 R primers in a PCR on genomic DNA. Clone 9 did not show the proper 3′ genomic structure.

To determine whether expression of WC-1 in heterokaryons containing the integrated *bar*::P*_tcu-1_*::*wc-1* construct was dependent on copper availability, protein extracts from strains grown in 200 µM CuSO_4_ or 200 µM BCS (plus 50 µM CuSO_4_) were examined by Western blot (Figure S3C). Expression of WC-1 in a control strain (Ø) and in clone #9 was independent of copper availability. Clones 1 and 7 showed clear differential regulation of WC-1 in high copper *vs.* BCS. We crossed clone 7 as described in the *Materials and Methods* to obtain a homokaryotic P*_tcu-1_wc-1* strain.

**Figure 4:**
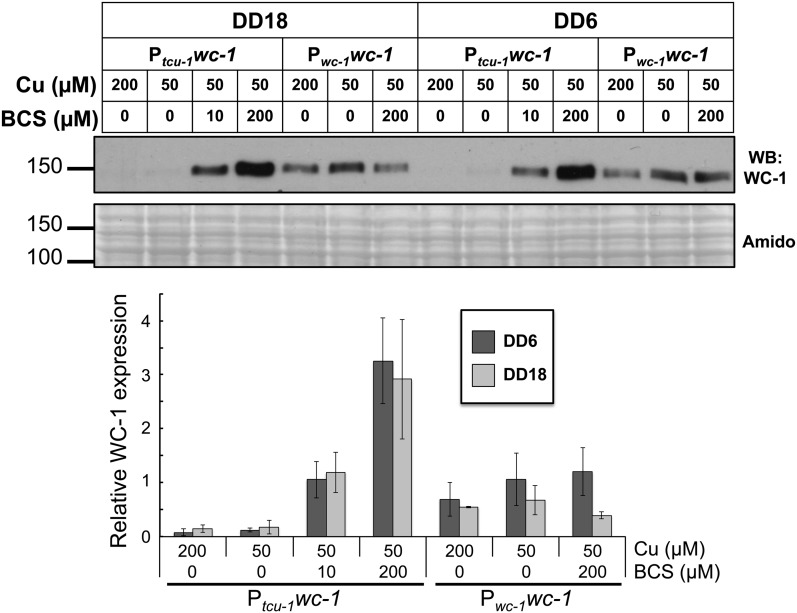
Effects of copper and BCS on P*_tcu-1_*-driven WC-1 protein production. Levels of WC-1 protein were analyzed by western blot (WB) on extracts from P*_wc-1_wc-1* and P*_tcu-1_wc-1* strains treated with copper and/or BCS as indicated for 6 hr. Extracts were generated from tissue grown for a total of 24 hr with 18 hr (DD18) or 6 hr (DD6) of that time in the dark at 25. The amido black staining (Amido) of the membrane in the lower panel demonstrates equal protein loading. Protein size markers (S) are shown, with the molecular weight (kDa) as indicated. Relative WC-1 expression (±SD) from two biological replicates is plotted below.

To determine the efficiency of P*_tcu-1_*-driven WC-1 control, the effects of Cu and BCS on WC-1 protein levels was assayed in the P*_tcu-1_wc-1* strain. Our ultimate goal was to test how P*_tcu-1_* control of WC-1 affects rhythms in conidial development in DD (a condition in which the clock is functional). Therefore, the levels of WC-1 protein were first examined in two time points in the dark (DD18, representing subjective afternoon and DD6, representing subjective late night). Figure 4 shows an inverse correlation between copper availability and WC-1 protein in the P*_tcu-1_wc-1* strain, which is independent of the time in DD. WC-1 protein levels in a P*_wc-1_wc-1* strain were unaffected by copper availability, and were not strongly influenced by the time in DD. Although it has been reported that WC-1 protein levels peak in abundance around DD6 and display a trough around DD18 (Lee *et al.* 2000), the amplitude of this rhythm can be quite low (Cheng *et al.* 2001), so our failure to observe a clear time of day difference in just two time points is not surprising. Finally, the WC-1 expression in the P*_tcu-1_wc-1* strain at 10 µM BCS most closely matched expression of the native P*_wc-1_wc-1* strain.

Rhythms in conidial development are typically assayed on medium with 0.1% glucose as a carbon source (Liu *et al.* 1997). We were worried that the WC-1 protein might not be fully induced under these conditions given the weaker response of *tcu-1* expression to BCS in 0.01% glucose medium (Figure 2). Therefore, we examined the effects of copper concentration on WC-1 protein levels in the P*_tcu_*_-1_*wc-1* strain at 0.1% and 2% glucose in two time points in DD (DD6 and DD18) and LL (Figure S4). WC-1 was well controlled by copper availability in both glucose concentrations, and independent of the light conditions.

**Figure 5:**
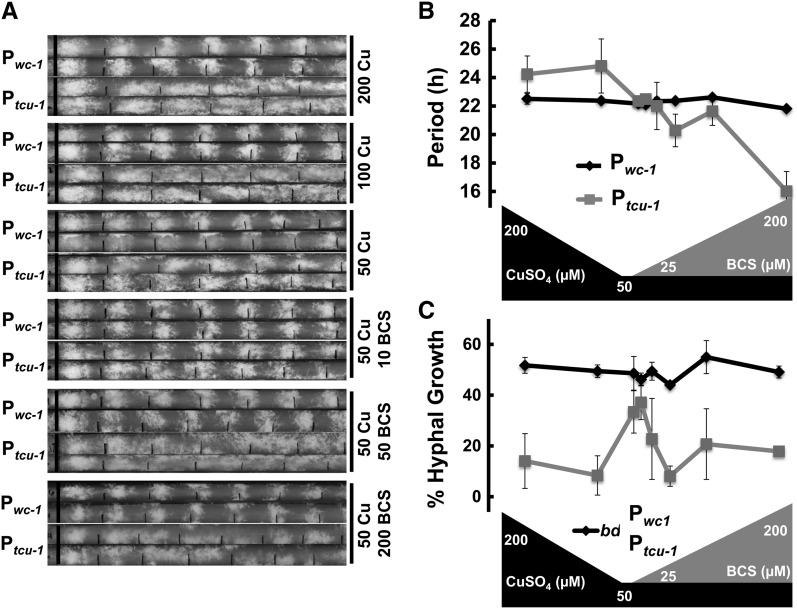
Effects of P*_tcu-1_*-controlled *wc-1* on circadian rhythms. (A) P*_wc-1_wc-1* and P*_tcu-1_wc-1* strains (denoted here, P*_wc-1_* and P*_tcu-1_*) were inoculated on race tubes containing the indicated concentrations of CuSO_4_ (Cu in µM) and BCS (BCS in µM), grown in LL for 24 hr at 30°, and then shifted to DD at 25°. The solid black line on the left marks the growth front at the time of the shift. Every 24 hr the growth front was marked under safe red lights (smaller tick marks). Two independent tubes are shown for each strain and condition. (B) The period of the conidiation rhythm *vs.* the Cu/BCS levels is plotted for P*_wc-1_wc-1* and P*_tcu-1_wc-1* strains. Each data point is the average period calculated from at least three race tubes (±SEM). (C) Percent of growth that is linear hyphae (and not aerial hyphae/conidia) is plotted *vs.* the Cu/BCS levels for P*_wc-1_wc-1* and P*_tcu-1_wc-1* strains.

Finally, to assess growth and rhythmicity, P*_wc-1_wc-1* and P*_tcu-1_wc-1* strains were inoculated on race tubes containing 200, 100, and 50 µM final CuSO_4_, or 50 µM CuSO_4_ with 10, 25, 50, 100, and 200 µM BCS (Figure 5A). Growth rates of both strains were only very mildly affected by changes in the availability of CuSO_4_ (Figure S5), suggesting that copper toxicity or starvation is not achieved under these conditions. The P*_wc-1_wc-1* strain grew ~50% of the time as linear hyphae compared with conidiating aerial hyphae, and developed bands of conidia with a period of ~22.3 hr independent of copper availability (Figure 5, B and C). In high copper, the P*_tcu-1_wc-1* strain was largely arrhythmic with conidiation predominating (only ~10% of the growth was linear hyphae), and had a long period, when a period could be measured (~24.5 hr), as expected for strains with reduced or no expression of WC-1 (Lee *et al.* 2003). As copper was reduced and BCS increased, normal rhythmicity was restored at 50 µM CuSO_4_ with or without 10 µM BCS. Above this level of BCS, rhythmicity became unreliable, and the oscillations accelerated (shorter period), as expected for strains overexpressing WC-1 (Cheng *et al.* 2001).

Together, these data demonstrate that the effects of copper availability on rhythmicity of the P*_tcu-1_wc-1* strain correlated with the protein expression levels of WC-1. Increased WC-1 expression accelerated, and decreased WC-1 expression decelerated, oscillations. When copper-controlled WC-1 expression matched native gene expression, circadian oscillations mimicked native rhythmicity and period on race tubes.

### Copper repression of an essential gene, *hpt-1*, phenocopies inviabiliy

To test whether P*_tcu-1_* is suitable to knock down expression of an essential gene and recapitulate loss of viability, we inserted P*_tcu-1_* into the 5′ region of the essential gene *hpt-1*, which encodes the sole histidine phospho-transferase in *N. crassa* (Banno *et al.* 2007; Borkovich *et al.* 2004). The primers used for this construction are listed in Table S2. Figure S6 shows the expression of HPT-1-FLAG in the control P*_hpt-1_hpt-1* strain, as well as in several P*_tcu-1_hpt-1* primary transformants after growth in 250 µM CuSO_4_ and 200 µM BCS. Four of six glufosinate-resistant transformants had the expected copper regulation of HPT-1-FLAG. A homokaryotic P*_tcu-1_hpt-1* strain was isolated from transformant #6 as described in the *Material and Methods*.

**Figure 6:**
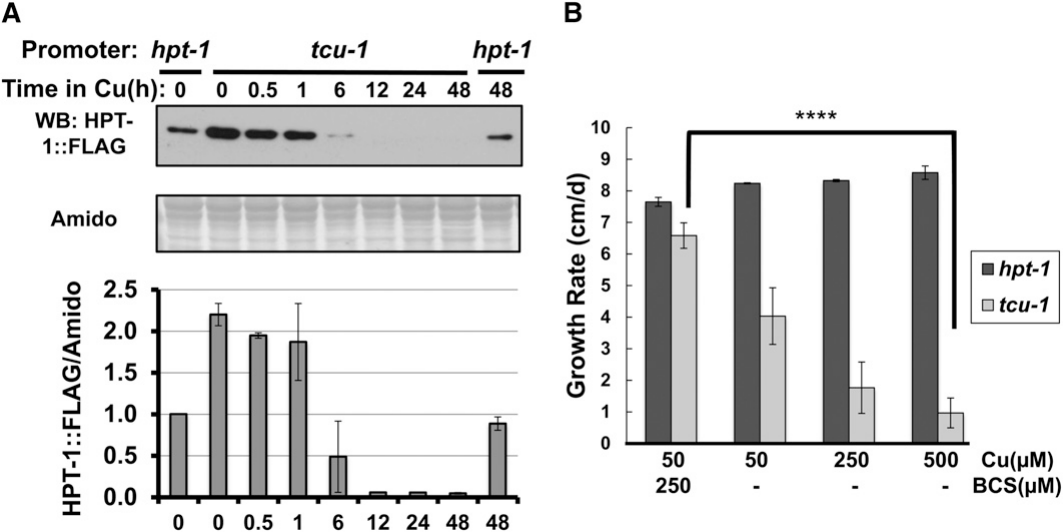
Effects of copper on HPT-1 protein levels and viability of in a P*_tcu-1_hpt-1* strain. (A) Strains with *hpt-1*::FLAG expression driven by the native (*hpt-1*) or copper promoters (*tcu-1*) were treated with 100 µM BCS for 10 hr, subsequently treated with 250 µM CuSO_4_ for the indicated times, and protein extracts were analyzed by Western blot for HPT-1::FLAG. The amido black staining (Amido) of the membrane in the lower panel demonstrates equal protein loading. This experiment was performed twice (N = 2), and the average HPT-1::FLAG/Amido signal (±SD) is plotted below. Expression from the native promoter at time 0 was set to 1 for each experiment. (B) The average growth rate (cm/d ± SD) of P*_tcu-1_ hpt-1* (*tcu-1*, light bars) and P*_hpt-1_hpt-1* (*hpt-1*, dark bars) strains is plotted *vs.* the copper/BCS content as indicated below. A significant difference (****T-test, *P* < 0.0004) in the growth rate of the P*_tcu-1_hpt-1* strain at low (250 µM BCS) *vs.* high copper (500 µM CuSO_4_), but not in the strain driven by the native promoter (P*_hpt-1_hpt-1*), is shown.

To examine how rapidly CuSO_4_ reduces HPT-1 protein in the P*_tcu-1_hpt-1* strain, BCS-induced cultures were treated with 250 µM CuSO_4_ for varying lengths of time, and the protein was analyzed by Western blot (Figure 6A). Compared with the P*_hpt-1_hpt-1* control strain, HPT-1 protein was well induced by BCS in the P*_tcu-1_hpt-1* strain. However, after treatment with Cu for 6 hr, the level of HPT-1 protein was already below that of the control strain, and longer Cu treatments reduced HPT-1 to below the level of detection. Treatment of the control strain with Cu for 48 hr had no effect on the level of HPT-1 protein, as expected. Thus, copper repression of HPT-1 is efficient and long lived.

To determine whether copper repression of P*_tcu-1_hpt-1* could phenocopy an *hpt-1* deletion, which is known to be lethal (Banno *et al.* 2007), race tubes containing CuSO_4_ and/or BCS were inoculated with P*_hpt-1_hpt-1* and P*_tcu-1_hpt-1* strains and allowed to grow at 30° for 3 d. The growth rate of the P*_hpt-1_hpt-1* strain was largely unaffected by the copper/BCS content of the race tubes, while the growth rate of the P*_tcu-1_hpt-1* strain was strongly inhibited by increasing copper content as predicted (Figure 6B). The growth rate of the P*_tcu-1_hpt-1* strain was low but constant over the course of several days, suggesting that HPT-1 protein was depleted but not completely abolished.

## Discussion

We have developed a system to control expression of any desired target gene in *N. crassa* by copper availability that will be beneficial for studies of gene function, including for example, overexpression of any transcription factor to identify its direct targets by chromatin immunoprecipitation-sequencing. This system relies on homologous recombination of split marker PCR-generated fragments, and simple addition of BCS (a copper chelator) or CuSO_4_ to tune P*_tcu-1_*-driven expression up or down, respectively (Figure 1). Activation is long lasting (Figure 1C), with the kinetics of activation varying according to the BCS concentration (Figure 1A). Lower levels of BCS cause slower activation, whereas greater levels of BCS result in more rapid activation. Repression is very rapid, and long lasting (Figure 1, B and C). Expression from P*_tcu-1_* is exquisitely sensitive to copper availability and insensitive to many other environmental insults, highlighting the strict specificity of copper expression control (Figure 2).

The P*_tcu-1_* system is versatile in that only a single strain needs to be generated for both overexpression and repression. Levels of induction are excellent, with P*_tcu-1_* inducing a large protein to levels that were significantly higher than the P*_qa-2_* system. The addition of BCS overexpressed the large WC-1 (150 kDa) protein at levels around three times greater than the native expression, resulting in the cultures becoming arrhythmic (Figures 4 and 5). In contrast, the *qa-2* system was unable to achieve expression above native levels at the greatest level of inducer, and these cultures had a WT period (Cheng *et al.* 2001). Overexpression of the smaller HPT-1::FLAG protein (23 kDa) driven by P*_tcu-1_* was even more efficient, with greater than 10-fold induction over WT (Figure S6). Furthermore, turn down of *wc-1* and *hpt-1* expression with the addition of copper phenocopied their respective deletion strains.

Although the study of essential genes can be difficult, this P*_tcu-1_* system provides the flexibility of gene expression control. It allows the growth of strains with an essential gene under P*_tcu-1_* control in the presence of BCS and low copper, but then permits the study of loss of function by increasing copper levels. In our experiments with the essential gene *hpt-1* we found that increasing copper levels drastically inhibited growth, thus recapitulating inviability.

Even though the P*_tcu-1_* system is highly specific, it does display some level of glucose-dependent repression, despite the contrary findings of a genome wide study (Xie *et al.* 2004). Our data on *tcu-1* itself, and on P*_tcu-1_* control of the *wc-1* and *hpt-1* genes suggest that complete repression of gene expression requires elevated glucose levels. For example, Figure 2 shows that in 0.01% (but not 2%) glucose, *tcu-1* mRNA was detectable even in the presence of added copper (C). Similarly, Figure S4 shows that P*_tcu-1_* driven WC-1 protein levels were greater in 0.1% than 2% glucose in the copper treated samples. Finally, copper suppression of P*_tcu-1_ hpt-1* strain growth required 2% glucose (data not shown). Thus, full suppression of gene expression by P*_tcu-1_* requires concomitant glucose repression. Taken together, P*_tcu-1_* is a valuable new tool for gene over-expression and turn-off, with the most complete turn-off accomplished in glucose-repressed conditions.

## Supplementary Material

Supporting Information
