# Supplementary material for: Regulation of Gene Expression in Neurospora crassa with a Copper Responsive Promoter
Source: G3 (Bethesda). 2013 Oct 18;3(12):2273–80. doi: 10.1534/g3.113.008821 (PMC3852388; doi:10.1534/g3.113.008821)
Supplement: Supporting Information [file supp_3_12_2273__index.html]

Regulation of Gene Expression in Neurospora crassa with a Copper Responsive Promoter — Supporting Information 

# Regulation of Gene Expression in *Neurospora crassa* with a Copper Responsive Promoter

## Supporting Information for Lamb, Vickery, and Bell-Pedersen, 2013

**Files in this Data Supplement:**

- Supporting Information - Figures S1-S6 and Tables S1-S2 (PDF, 865 KB)
- Figure S1 - Copper levels control expression of the *N. crassa* high affinity copper transporter (*tcu-1*) gene in a WT strain. (PDF, 437 KB)
- Figure S2 - Quantitation of *tcu-1* expression after induction by BCS. (PDF, 524 KB)
- Figure S3 - Integration of P*tcu-1* into the *wc-1* locus. (PDF, 429 KB)
- Figure S4 - Effects of glucose concentration on P*tcu-1* driven WC-1 protein. (PDF, 459 KB)
- Figure S5 - Effects of copper and BCS on growth. (PDF, 410 KB)
- Figure S6 - Effects of copper and BCS on P*tcu-1* driven HPT1-FLAG protein production. (PDF, 410 KB)
- Table S1 - Primers used for P*tcu-1**wc-1* strain construction. (PDF, 396 KB)
- Table S2 - Primers used for P*tcu-1**hpt-1* strain construction. (PDF, 395 KB)
