# Supplementary material for: Regulation of Gene Expression in Neurospora crassa with a Copper Responsive Promoter
Source: G3 (Bethesda). 2013 Oct 18;3(12):2273–80. doi: 10.1534/g3.113.008821 (PMC3852388; doi:10.1534/g3.113.008821)
Supplement: Supporting Information [file supp_g3.113.008821_008821SI.pdf]

G3: Genes | Genomes | Genetics

Regulation of Gene Expression in *Neurospora crassa* with a Copper Responsive Promoter

Teresa M Lamb, Justin Vickery, and Deborah Bell-Pedersen[#]

Department of Biology, Texas A&M University, College Station, TX 77843

[#] Corresponding author.
Mailing address: Department of Biology, TAMU 3258, College Station, TX 77843.
Phone: (979) 847-9237
Fax: (979) 845-2891
E-mail: dpedersen@bio.tamu.edu

DOI: 10.1534/g3.113.008821

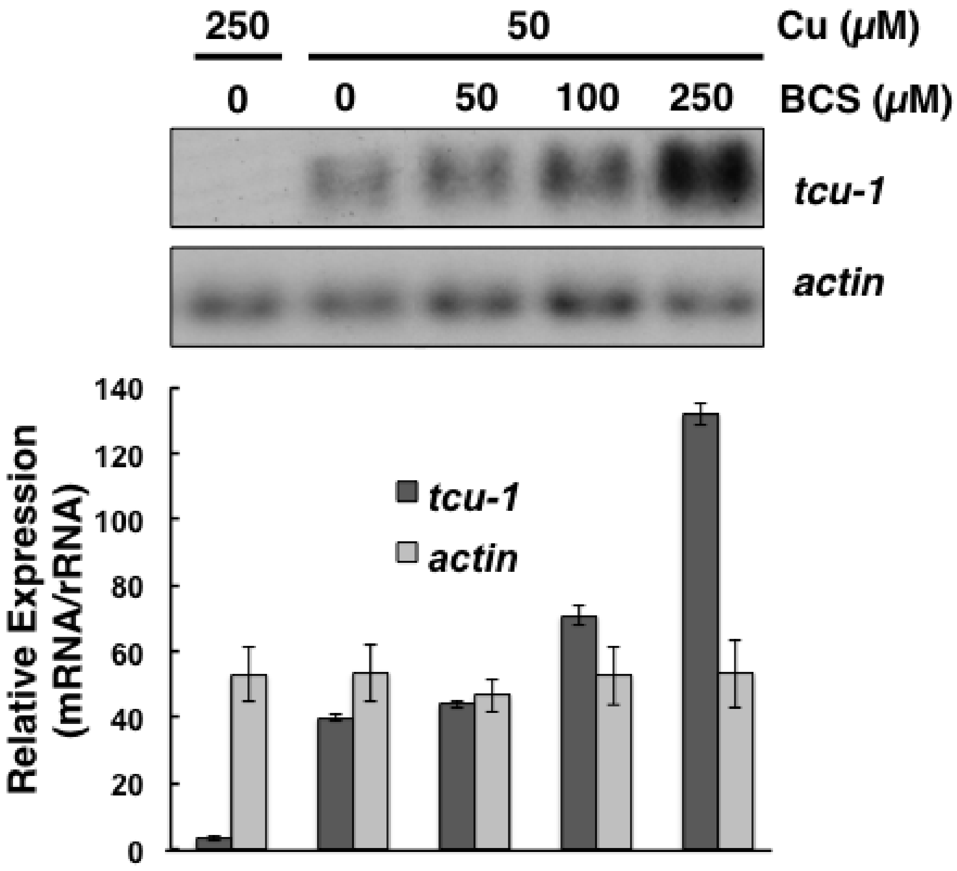

Figure S1 Copper levels control expression of the *N. crassa* high affinity copper transporter (*tcu-1*) gene in a WT strain. Expression of *tcu-1* and *actin* after 8 h of treatment with 250 μM $CuSO_4$ (Cu) or 0, 50, 100, 250 μM BCS (in media containing 50 μM $CuSO_4$) was determined by Northern analysis. Quantitation of three independent experiments is shown below (+/- SD), where the relative expression was calculated as the ratio of *tcu-1* or *actin* specific signal to rRNA (not shown).

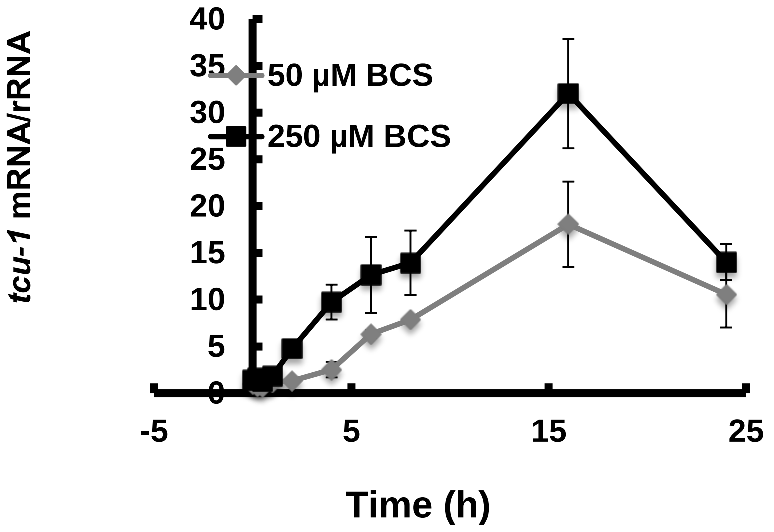

Figure S2 Quantitation of *tcu-1* expression after induction by BCS. The average density of the *tcu-1* signal divided by the rRNA signal (N=2 +/- SD) was plotted versus time for the indicated concentrations of BCS. The *tcu-1* mRNA/rRNA value at time zero was normalized to one.

Table S1 Primers used for $P_{tcu\text{-}1}$*wc-1* strain construction.

Primer Name	5' end tail	3' end homology
WC1 P1 F		CATTGCAATGCCCTCATTTTGG
WC1 P2 R	TTAGGTCGAC	CGGTCGACGAGTGACGTTG
WC1 P3 F	TCGTCGACCG	GTCGACCTAAATCTCGGTGAC
WC1 P6 R	TGTTGTTCAT	GGTTGGGGATGTGTGTGCGA
WC1 P7 F	ATCCCCAACC	ATGAACAACAACTACTACGGTTC
WC1 P8 R		GAGCCACTATCCATGTTCATGT
WC1 P9 F		GATCATGGGCGACGGTGGAAAA
WC1 P10 R		CACCTCCACCAAAGCCCTGCTC

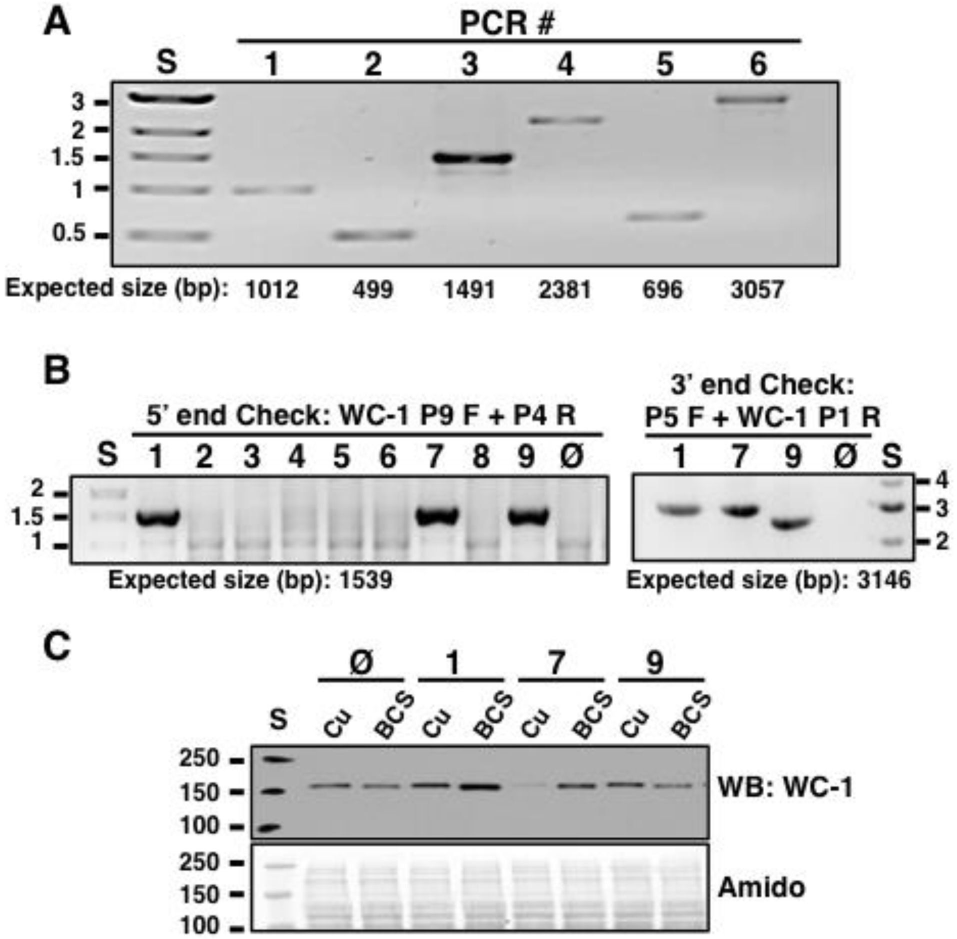

Figure S3 Integration of P_{tcu-1} into the *wc-1* locus. (**A**) Agarose gel electrophoresis of the PCR products that generate the 5' and 3' integrating fragments for the *wc-1* locus knock in. The PCR # relates to the scheme shown in Figure 4B. The expected size of each PCR product is listed below, and DNA size markers (S, in kbp) are shown on the left (**B**) Agarose gel electrophoresis of the products generated by PCR using the indicated primers to test the genomic DNA configuration (5' end and 3' end) of control (Ø) and transformed (1-9) strains. The expected size of the PCR product is listed below, and DNA size markers (S, in kbp) are shown for each gel. (**C**) Test of Cu (250 µM) and BCS (250 µM) responsiveness of control (Ø) and transformed strains (1, 7 and 9) by WC-1 Western blot (WB). The amido black staining (Amido) of the membrane in the lower panel demonstrates equal protein loading. Protein size markers (S) are shown, with the molecular weight (kDa) as indicated.

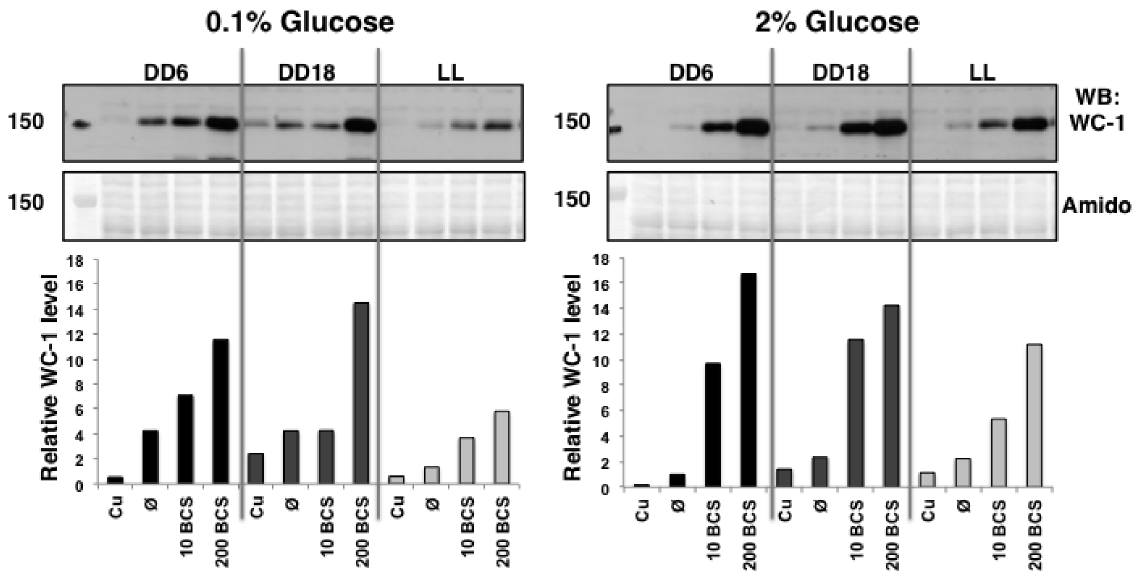

Figure S4 Effects of glucose concentration on P_{tcu-1} driven WC-1 protein. Levels of WC-1 protein were analyzed by Western blot (WB) on extracts from the P_{tcu-1}*wc-1* strain grown under low (0.1%) and high (2%) glucose conditions, and then treated with nothing (Ø), 200 µM copper (Cu) and/or BCS (in µM) as indicated for 6 h. Extracts were generated from tissue grown for a total of 24 h, with 18 h (DD18) or 6 h (DD6) of that time in the dark or in LL at 25°C. The amido black staining (Amido) of the membrane in the lower panel demonstrates equal protein loading. Protein size markers (S) are shown, with the molecular weight (kDa) as indicated. The relative expression level (WC-1 protein/total protein signal) is plotted below.

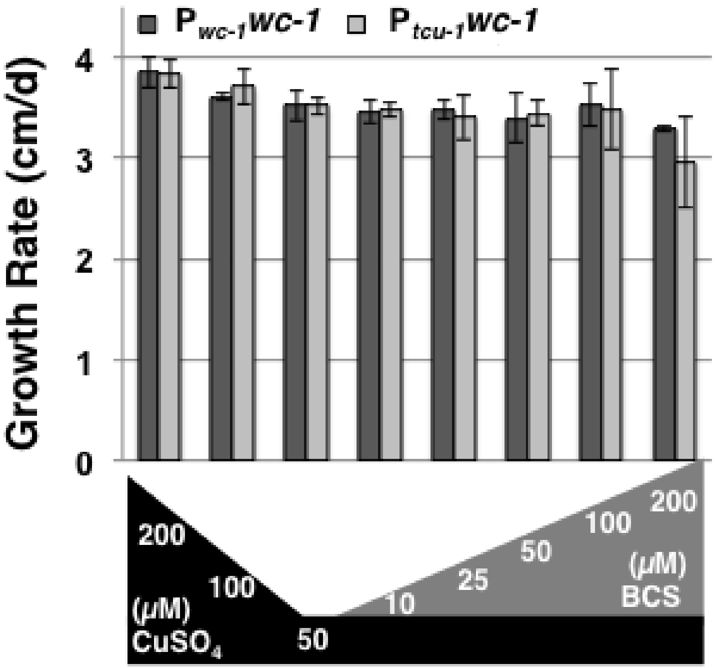

Figure S5 Effects of copper and BCS on growth. The average growth rate of $P_{wc\text{-}1}$ *wc-1* and $P_{tcu\text{-}1}$ *wc-1* strains is plotted versus the copper and BCS media content as indicated. Data represent the average growth rate ± SD of a minimum N=3 race tubes per strain and condition.

Table S2 Primers used for P_{tcu-1}*hpt-1* strain construction.

Primer Name	5' end tail	3' end homology
HPT1 P1 F		TTGACGGCCTGTACATGTAAGT
HPT1 P2 R	TTAGGTCGAC	CGCTCGTGGTTAGTTGCCGA
HPT1 P3 F	ATCAAGACATA	GTCGACCTAAATCTCGGTGAC
HPT1 P6 R	AATCCGGCAT	GGTTGGGGATGTGTGTGCGA
HPT1 P7 F	ATCCCCAACC	ATGCCGGATTTCGGAGAGCAC
HPT1 P8 R		AACATCATGTCCGCCAGCACG
HPT1 P9 F		TTCCAGCATCTGCTCTGAACC
HPT1 P10 R		CTGGCTGGAAGAAGCCGTGTG

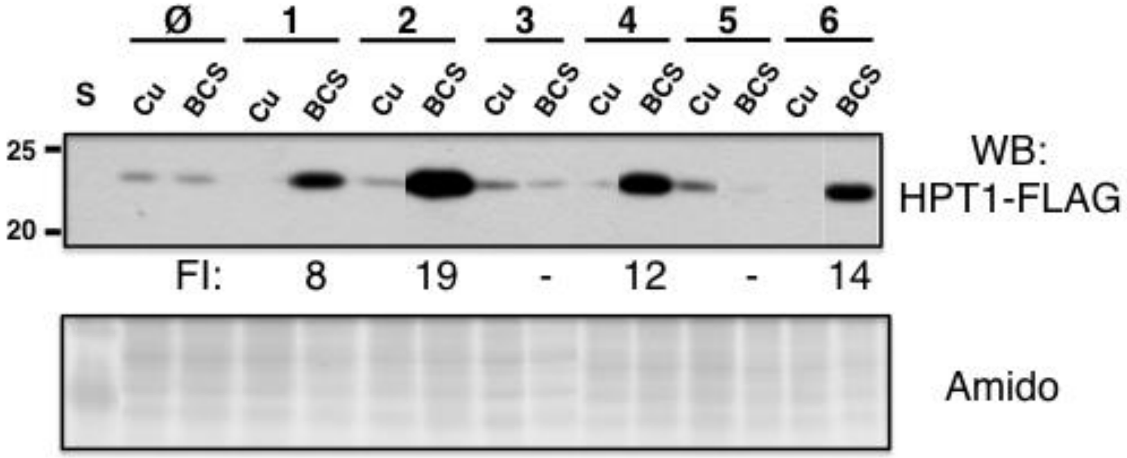

Figure S6 Effects of copper and BCS on P_{tcu-1} driven HPT1-FLAG protein production. 100 µg of total protein extract from control P_{hph-1}*hpt-1*(Ø) and P_{tcu-1}*hpt-1* transformed strains (1-6) grown in Cu (250 µM) and BCS (200 µM), were analyzed by Western blot for HPT-1::FLAG immuno-reactivity. While HPT-1-FLAG expression in the control strain does not respond to copper availability, expression in transformants #1, #2, #4, and #6 clearly increased in the presence of the copper chelator, BCS. Relative intensities of the HPT-1-FLAG signal normalized by the amido black staining were calculated, and the fold induction (FI) of HPT-1-FLAG in these transformants over the WT strain is shown below (for the BCS treatments). The lower panel demonstrates even protein loading by amido black staining (Amido).
