# Supplementary material for: Regulation of Gene Expression in Neurospora crassa with a Copper Responsive Promoter
Source: G3 (Bethesda). 2013 Oct 18;3(12):2273–80. doi: 10.1534/g3.113.008821 (PMC3852388; doi:10.1534/g3.113.008821)
Supplement: Supporting Information [file supp_g3.113.008821_FigureS1.pdf]

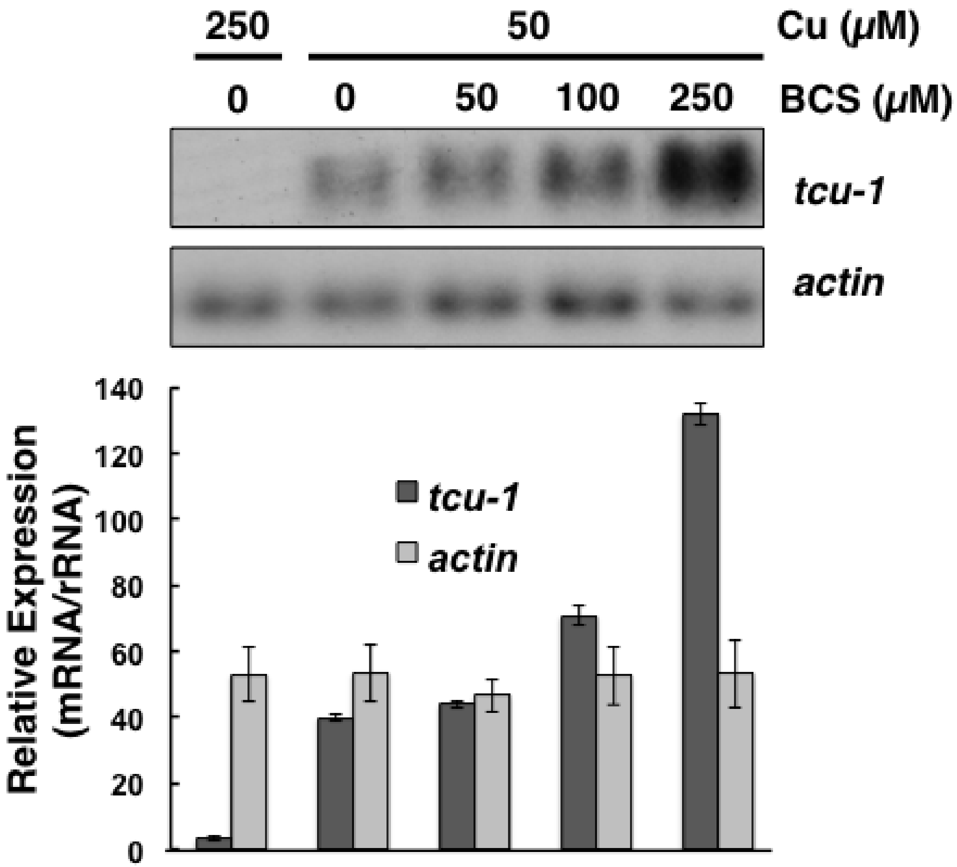

Figure S1 Copper levels control expression of the *N. crassa* high affinity copper transporter (*tcu-1*) gene in a WT strain. Expression of *tcu-1* and *actin* after 8 h of treatment with 250 μM $CuSO_4$ (Cu) or 0, 50, 100, 250 μM BCS (in media containing 50 μM $CuSO_4$) was determined by Northern analysis. Quantitation of three independent experiments is shown below (+/- SD), where the relative expression was calculated as the ratio of *tcu-1* or *actin* specific signal to rRNA (not shown).
