# Supplementary material for: Regulation of Gene Expression in Neurospora crassa with a Copper Responsive Promoter
Source: G3 (Bethesda). 2013 Oct 18;3(12):2273–80. doi: 10.1534/g3.113.008821 (PMC3852388; doi:10.1534/g3.113.008821)
Supplement: Supporting Information [file supp_g3.113.008821_FigureS2.pdf]

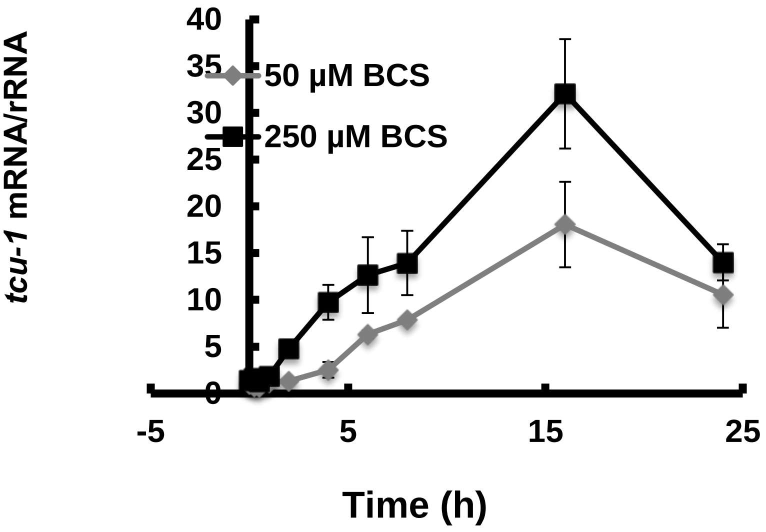

Figure S2 Quantitation of *tcu-1* expression after induction by BCS. The average density of the *tcu-1* signal divided by the rRNA signal (N=2 +/- SD) was plotted versus time for the indicated concentrations of BCS. The *tcu-1* mRNA/rRNA value at time zero was normalized to one.
