# Supplementary material for: Regulation of Gene Expression in Neurospora crassa with a Copper Responsive Promoter
Source: G3 (Bethesda). 2013 Oct 18;3(12):2273–80. doi: 10.1534/g3.113.008821 (PMC3852388; doi:10.1534/g3.113.008821)
Supplement: Supporting Information [file supp_g3.113.008821_FigureS3.pdf]

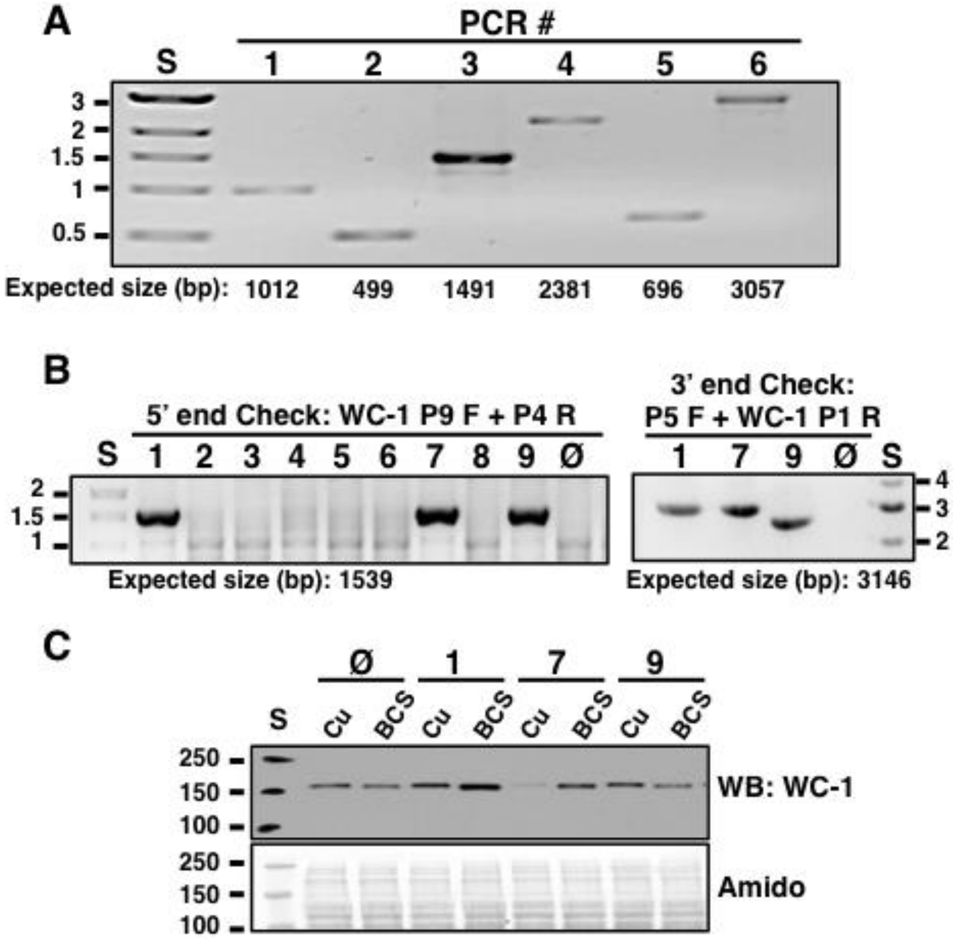

Figure S3 Integration of P_{tcu-1} into the *wc-1* locus. (**A**) Agarose gel electrophoresis of the PCR products that generate the 5' and 3' integrating fragments for the *wc-1* locus knock in. The PCR # relates to the scheme shown in Figure 4B. The expected size of each PCR product is listed below, and DNA size markers (S, in kbp) are shown on the left (**B**) Agarose gel electrophoresis of the products generated by PCR using the indicated primers to test the genomic DNA configuration (5' end and 3' end) of control (Ø) and transformed (1-9) strains. The expected size of the PCR product is listed below, and DNA size markers (S, in kbp) are shown for each gel. (**C**) Test of Cu (250 µM) and BCS (250 µM) responsiveness of control (Ø) and transformed strains (1, 7 and 9) by WC-1 Western blot (WB). The amido black staining (Amido) of the membrane in the lower panel demonstrates equal protein loading. Protein size markers (S) are shown, with the molecular weight (kDa) as indicated.
