# Supplementary material for: Regulation of Gene Expression in Neurospora crassa with a Copper Responsive Promoter
Source: G3 (Bethesda). 2013 Oct 18;3(12):2273–80. doi: 10.1534/g3.113.008821 (PMC3852388; doi:10.1534/g3.113.008821)
Supplement: Supporting Information [file supp_g3.113.008821_FigureS4.pdf]

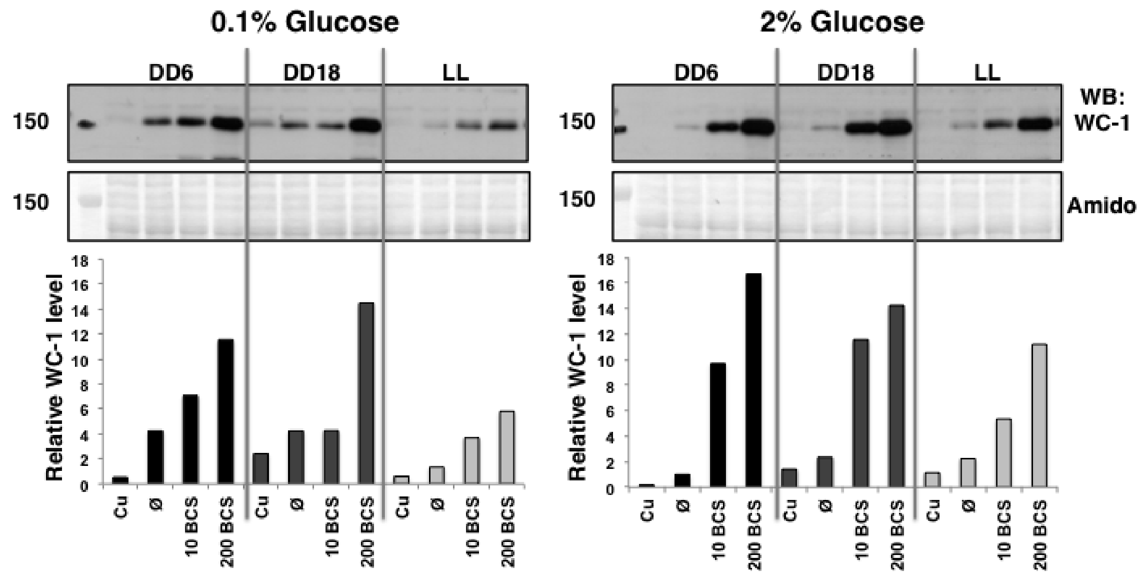

Figure S4 Effects of glucose concentration on $P_{tcu\text{-}1}$ driven WC-1 protein. Levels of WC-1 protein were analyzed by Western blot (WB) on extracts from the $P_{tcu\text{-}1}$*wc-1* strain grown under low (0.1%) and high (2%) glucose conditions, and then treated with nothing (Ø), 200 µM copper (Cu) and/or BCS (in µM) as indicated for 6 h. Extracts were generated from tissue grown for a total of 24 h, with 18 h (DD18) or 6 h (DD6) of that time in the dark or in LL at 25°C. The amido black staining (Amido) of the membrane in the lower panel demonstrates equal protein loading. Protein size markers (S) are shown, with the molecular weight (kDa) as indicated. The relative expression level (WC-1 protein/total protein signal) is plotted below.
