# Supplementary material for: Regulation of Gene Expression in Neurospora crassa with a Copper Responsive Promoter
Source: G3 (Bethesda). 2013 Oct 18;3(12):2273–80. doi: 10.1534/g3.113.008821 (PMC3852388; doi:10.1534/g3.113.008821)
Supplement: Supporting Information [file supp_g3.113.008821_FigureS5.pdf]

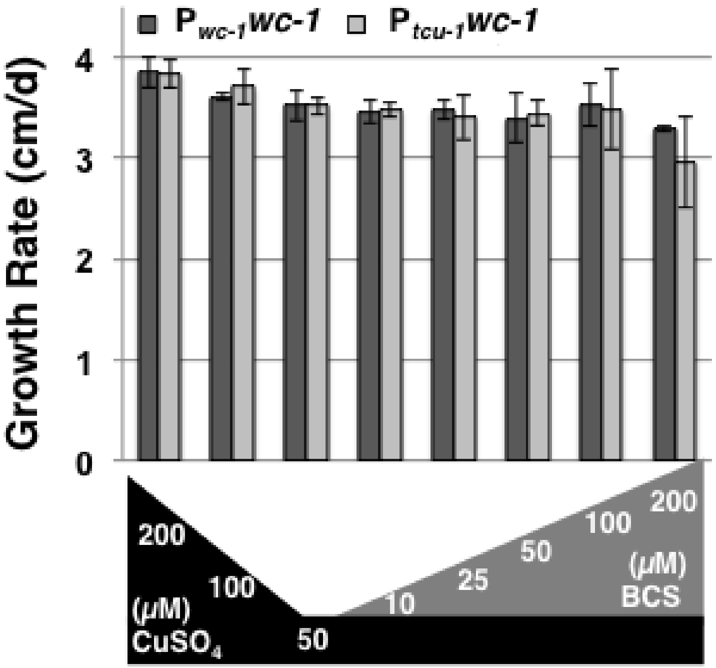

Figure S5 Effects of copper and BCS on growth. The average growth rate of $P_{wc\text{-}1}$ *wc-1* and $P_{tcu\text{-}1}$ *wc-1* strains is plotted versus the copper and BCS media content as indicated. Data represent the average growth rate ± SD of a minimum N=3 race tubes per strain and condition.
