# Supplementary material for: Regulation of Gene Expression in Neurospora crassa with a Copper Responsive Promoter
Source: G3 (Bethesda). 2013 Oct 18;3(12):2273–80. doi: 10.1534/g3.113.008821 (PMC3852388; doi:10.1534/g3.113.008821)
Supplement: Supporting Information [file supp_g3.113.008821_FigureS6.pdf]

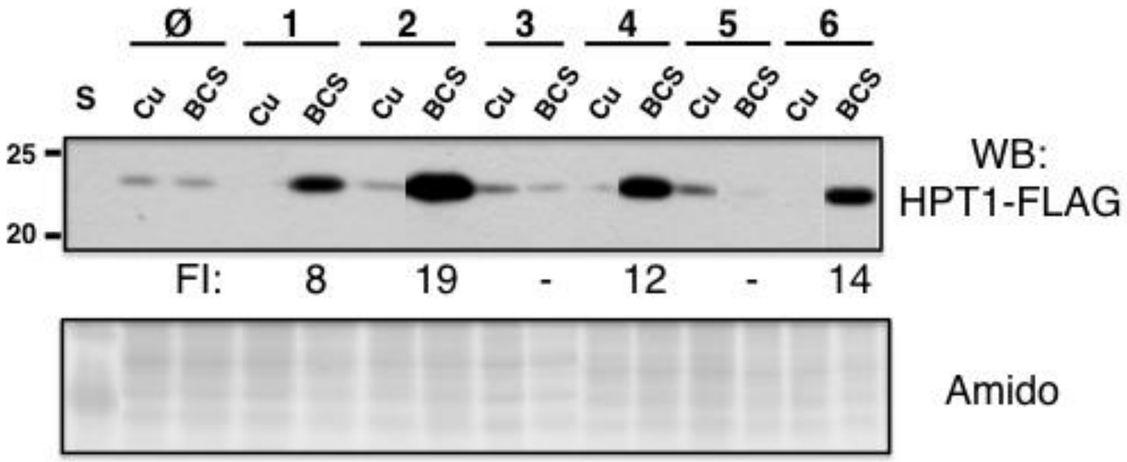

Figure S6 Effects of copper and BCS on P_{tcu-1} driven HPT1-FLAG protein production. 100 µg of total protein extract from control P_{hph-1}*hpt-1*(Ø) and P_{tcu-1}*hpt-1* transformed strains (1-6) grown in Cu (250 µM) and BCS (200 µM), were analyzed by Western blot for HPT-1::FLAG immuno-reactivity. While HPT-1-FLAG expression in the control strain does not respond to copper availability, expression in transformants #1, #2, #4, and #6 clearly increased in the presence of the copper chelator, BCS. Relative intensities of the HPT-1-FLAG signal normalized by the amido black staining were calculated, and the fold induction (FI) of HPT-1-FLAG in these transformants over the WT strain is shown below (for the BCS treatments). The lower panel demonstrates even protein loading by amido black staining (Amido).
