# Supplementary material for: Regulation of Gene Expression in Neurospora crassa with a Copper Responsive Promoter
Source: G3 (Bethesda). 2013 Oct 18;3(12):2273–80. doi: 10.1534/g3.113.008821 (PMC3852388; doi:10.1534/g3.113.008821)
Supplement: Supporting Information [file supp_g3.113.008821_TableS1.pdf]

Table S1 Primers used for P$_{tcu\text{-}1}$*wc-1* strain construction.

Primer Name	5' end tail	3' end homology
WC1 P1 F		CATTGCAATGCCCTCATTTTGG
WC1 P2 R	TTAGGTCGAC	CGGTCGACGAGTGACGTTG
WC1 P3 F	TCGTCGACCG	GTCGACCTAAATCTCGGTGAC
WC1 P6 R	TGTTGTTCAT	GGTTGGGGATGTGTGTGCGA
WC1 P7 F	ATCCCCAACC	ATGAACAACAACTACTACGGTTC
WC1 P8 R		GAGCCACTATCCATGTTCATGT
WC1 P9 F		GATCATGGGCGACGGTGGAAAA
WC1 P10 R		CACCTCCACCAAAGCCCTGCTC
