# Supplementary material for: Regulation of Gene Expression in Neurospora crassa with a Copper Responsive Promoter
Source: G3 (Bethesda). 2013 Oct 18;3(12):2273–80. doi: 10.1534/g3.113.008821 (PMC3852388; doi:10.1534/g3.113.008821)
Supplement: Supporting Information [file supp_g3.113.008821_TableS2.pdf]

Table S2 Primers used for P_{tcu-1}*hpt-1* strain construction.

Primer Name	5' end tail	3' end homology
HPT1 P1 F		TTGACGGCCTGTACATGTAAGT
HPT1 P2 R	TTAGGTCGAC	CGCTCGTGGTTAGTTGCCGA
HPT1 P3 F	ATCAAGACATA	GTCGACCTAAATCTCGGTGAC
HPT1 P6 R	AATCCGGCAT	GGTTGGGGATGTGTGTGCGA
HPT1 P7 F	ATCCCCAACC	ATGCCGGATTTCGGAGAGCAC
HPT1 P8 R		AACATCATGTCCGCCAGCACG
HPT1 P9 F		TTCCAGCATCTGCTCTGAACC
HPT1 P10 R		CTGGCTGGAAGAAGCCGTGTG
